# Development and Validation of a HPLC-UV Method for the Evaluation of Ellagic Acid in Liquid Extracts of* Eugenia uniflora* L. (Myrtaceae) Leaves and Its Ultrasound-Assisted Extraction Optimization

**DOI:** 10.1155/2017/1501038

**Published:** 2017-07-06

**Authors:** Paulo Isaac Dias Assunção, Edemilson Cardoso da Conceição, Leonardo Luiz Borges, Joelma Abadia Marciano de Paula

**Affiliations:** ^1^Campus Anápolis de Ciências Exatas e Tecnológicas, Universidade Estadual de Goiás, Caixa Postal 459, 75132-903 Anápolis, GO, Brazil; ^2^Laboratório de Pesquisa, Desenvolvimento & Inovação de Bioprodutos, Universidade Federal de Goiás, Caixa Postal 131, 74605-170 Goiânia, GO, Brazil

## Abstract

A simple HPLC-UV method has been developed and validated for the quantification of ellagic acid (EA) in ethanol extracts of* Eugenia uniflora *L. (Myrtaceae) leaves. The ultrasound-assisted extraction (UAE) optimization was performed using a Box Behnken design (3^3^) combined with response surface methodology to study the effects of the ethanol concentration (%, w/w), extraction time (minutes), and temperature (°C) on the EA concentration. The optimized results showed that the highest extraction yield of EA by UAE was 26.0 *μ*g mL^−1^ when using 44% (w/w) ethanol as the solvent, 22 minutes as the extraction time, and 59°C as the extraction temperature. The concentration of EA in relation to the predicted value was 93.7%  ±  0.4. UAE showed a strong potential for EA extraction.

## 1. Introduction

*Eugenia uniflora *L., popularly known as Surinam cherry, is one of more than 5,500 species from the Myrtaceae family [1]. The species is native to South America despite being quite widespread in other continents [2]. According to popular use, the infusion of the leaves is used as a diuretic, antirheumatic, and antipyretic as well as in the control of hypotension, blood cholesterol, and uric acid in the urine [3–6].

Some properties of* E. uniflora *leaves, such as their hypotensive activity, anti-inflammatory activity, and reduction of postprandial hyperglycemia, have already been verified by in vivo studies [4, 7, 8].

Many of the species of the genus* Eugenia* draw attention for their essential oil and tannin contents. In 1997, it was reported for the first time that eugeniflorin D1 and eugeniflorin D2 as ellagitannins were present in the methanol extract of the leaves of* E. uniflora *[9]. The authors also noted the presence of oenothein B. The antiviral and antiproliferative activities of these compounds were later demonstrated [10, 11].

From the hydrolysis of the ester bonds of ellagitannins, hexaidroxidifenoil units are formed, and these, in turn, undergo a process of spontaneous lactonization leading to the formation of ellagic acid (EA) [12]. EA is a phenolic compound with poor water solubility whose presence has already been reported in human plasma [13]. This compound is often associated with several biological activities, among which the antioxidant, anti-inflammatory, and antiproliferative activities stand out [13–18].

Various methods of chemical and biochemical analysis have been employed to determine tannins and their derivatives in different plant species [19–22]. However, the method of preparation of the material to evaluate these compounds has shown potential for further study and optimization techniques.

The method of ultrasound-assisted extraction (UAE) is approached as a modern extraction alternative [23]. Compared with the conventional extraction methods that involve heating and reflux, UAE offers an alternative with lower losses due to phenolic oxidation, hydrolysis, and ionization [24]. In view of other modern techniques, such as extraction in supercritical fluids and assisted by microwaves, UAE is considered to be a low-cost, simple, and efficient technique [25]. Its extraction mechanism is attributed to the cavitation phenomenon, the interaction of mechanical forces, and their thermal impact. This provides the disruption of the cell wall, thus reducing the particle size and increasing mass transfer across the cell membrane [26].

Improving the performance of a system, process, or product to obtain greater benefit can be understood by the concept of optimization [27]. In recent years, tools for multivariate analysis have been frequently used in the optimization of analytical methods mainly due to the reduction in the number of experiments and consequently in the research costs [28–31]. Moreover, these tools allow the development of mathematical models in the evaluation of the interactions between variables [32].

This work aimed to develop and validate a high-performance liquid chromatography (HPLC) analytical method for the quantification of EA in liquid extracts of* E. uniflora *leaves and optimize the UAE of this chemical marker.

## 2. Materials and Methods

### 2.1. Chemicals and Reagents

Ethanol 95% (v/v) (Vetec, Duque de Caxias, SP, Brazil) and water filtered through a Milli-Q apparatus (Millipore, Bellerica, MA, USA) were used to prepare the extraction solutions. Methanol, acetonitrile (J. T. Baker, Center Valley, PA, USA), and Milli-Q water were used in standard, sample, and mobile phase preparations. Formic acid, acetic acid, orthophosphoric acid, trifluoroacetic acid, and trichloroacetic acid (Sigma, St. Louis, MO, USA) were used as acidifying agents for the mobile phase. Ellagic acid, gallic acid, catechin, epicatechin, rutin, hesperidin, and quercetin were of analytical grade (Sigma, St. Louis, MO, USA) and used as external standards.

### 2.2. Equipment

The ultrasound-assisted extractions (UAE) were performed with an ultrasonic device Unique® USC 2800A, 40 kHz (Indaiatuba, SP, Brazil). A Varian HPLC ProStar® (Palo Alto, CA, USP) with a 240 pump, 310 sample manager, 20599 UV detector, and Star (version 6.2) software was used. Chromatographic separation was performed using a Supelco® C18 column (250 mm × 4.6 mm, 5 *μ*m). All of the solutions were filtered through a 0.45 *μ*m membrane (Merck, Bellerica, MA, USA). SPE was carried out on a C-18 Supelco cartridge (6.0 mL capacity).

### 2.3. Plant Materials

*E. uniflora * leaves were collected in June 2014 in the city of Anápolis, Goiás state, Brazil (16°17′13.8′′S and 48°57′22.7′′W, altitude 1.074 m). The authenticity of the material collected was verified by Dra. Myrlei Luciene dos Santos, and voucher specimens were deposited in the herbarium of the Universidade Estadual de Goiás with the registration number HUEG2090. The material was dried at room temperature and ground in a Wiley mill. The packaging was done in containers protected from light and moisture. The characterization of the powder obtained from the leaves of* E. uniflora* [33] revealed a moisture content of 0.2 ± 8.90%, a total ash content of 9.14%  ±  0.1, a swelling index of 4.16 ± 0.03 mL g^−1^ in water, and a particle size approximately 250 *μ*m.

### 2.4. Preliminary Tests for the HPLC-UV Method Development

For the election of a chemical marker, the evaluation of the chromatographic profile of the liquid extract from the leaves of* E. uniflora *was carried out. Extraction solutions were prepared in 70% ethanol (w/w) at a drug/solvent ratio of 15% (w/v) and kept in an ultrasound bath for 30 minutes at room temperature. The chromatographic conditions for the evaluation of the profile were adapted from the work of Kim et al. [34].

The identification of the constituents present in the chromatographic profile of the extract was carried out by comparison with the retention times (RT) of peaks in the standard solutions (gallic acid, ellagic acid, catechin, epicatechin, hesperidin, quercetin, and rutin at a concentration of 100 *μ*g mL^−1^). Among the compounds identified, EA was selected as the chemical marker to study the development of the analytical methodology.

Once the chemical marker was selected, three alcohol contents were evaluated for the extraction solution: 50, 70, and 90% ethanol (w/w). The preparation of the extracts was performed in triplicate for each of the solvents. The drug/solvent ratio was maintained at 15% (w/v), and the process was conducted in an ultrasound bath for 30 minutes at room temperature. The chromatographic evaluation of the extracts was performed using the same methodology adapted from Kim et al. [34].

### 2.5. Sample Preparation

Based on the data of the preliminary studies, the sample preparation for the development of the methodology used 50% ethanol (w/w) as the extraction solvent. The drug/solvent mixture was maintained at a ratio of 10% (w/v) to allow a better evaluation of the peak of the chemical marker, and the process was conducted in ultrasound equipment for 30 minutes at room temperature.

### 2.6. Solid Phase Extraction (SPE)

SPE was performed according to the method reported by Lopes et al. [35]. One milliliter of filtered extract was transferred to the SPE cartridge. Elution was accomplished with a mobile phase composed of water : acetonitrile (80 : 20). The eluted extract was collected in a 10 mL volumetric flask until the volume was completed.

### 2.7. Chromatographic Conditions

The chromatographic conditions for the evaluation of EA in the extracts were obtained after testing different methods reported in the literature and varying some of these conditions [34, 36]. We also evaluated isocratic elution with mobile phases constituted of water/acetonitrile or methanol; three different acidifying agents were tested (trichloroacetic acid, trifluoroacetic acid, and orthophosphoric acid), flows ranging from 0.5 to 1.2 mL min^−1^ and wavelength of 254 and 280 nm. Throughout the development of this study, the injection volume of the chromatographic system was kept constant at 10 *μ*L.

### 2.8. HPLC-UV Method Validation

The parameters and specifications for the method validation were determined based on the International Conference on the Harmonization (ICH) of Technical Requirements for the Registration of Pharmaceuticals for Human Use and the Brazilian legislation [37–40].

The system suitability was evaluated by six consecutive injections of the same sample solution. The following parameters were considered: number theoretical plates, *k*′, tailing, and relative standard deviation (RSD) of the peak areas of EA.

The selectivity was determined by comparing the chromatograms obtained with the standard solution, sample solution, mobile phase, and blank (water : methanol, 80 : 20, v/v), in the detection of interferents through coelution.

Linearity was determined by the analytical curves of the EA standard at five concentration levels (14.5, 29.3, 24.2, 29.0, and 33.8 *μ*g mL^−1^) in methanol. The analysis was performed in triplicate for each concentration level and injected into the chromatographic system. The linear and determination coefficients were calculated mathematically.

A calibration curve was prepared in triplicate with seven different concentrations of a standard solution of EA (6.4, 9.6, 12.8, 16.0, 19.2, 22.4, and 25.6 *μ*g mL^−1^) in methanol. The limit of detection (LOD) and limit of quantification (LOQ) were calculated from the three standard curves using the standard deviation (SD_*a*_) of the intercept with the *y*-axis and the slope of the calibration curve (*S*) according to

(1) LOD=SDa×3S,LOQ=SDa×10S.

Repeatability (intraday) and intermediate precision (interday) were evaluated. The relative standard deviation (RSD) was determined for twelve injections (two injections for each preparation). The intermediate precision was evaluated by this same process performed on a different day.

The accuracy of the method was measured through the analyte recovery test. Volumetric liquid extract solutions were prepared (80, 100, and 120% of the standard concentration in the linear range) in methanol with and without the addition of a known standard amount of EA (16.0 *μ*g mL^−1^). The accuracy was calculated for every level according to

(2) $$\begin{array}{c} \begin{array}{c} \text{Accuracy}=\frac{\text{sample conc. with standard}-\text{sample conc. without standard}}{\text{standard theoretical concentration}}\times 100. \end{array} \end{array}$$

The robustness of the method was evaluated by analyzing the results of the EA peak area of sample preparations obtained from both the original analysis conditions and modified conditions. The analysis was performed in triplicate and the results were evaluated by the RSD calculation. The following parameters were changed: mobile phase flow, injection volume, and column lot.

### 2.9. Ultrasound-Assisted Extraction Process

The optimization of the EA UAE process in leaves of* E. uniflora* was performed from the experiments set out in the Box Behnken design (3^3^) for the following parameters in the evaluation of the EA concentration: ethanol concentration (%, w/w), extraction time (minutes), and temperature (°C). The complete design including three replicates at the central point is presented in Table 1.

A second-order model was employed to express the effects of the independent variables on the EA concentration (see (3)).

(3) $$\begin{array}{c} y=\beta_{0}+\overset{k}{\underset{i=1}{\sum }}\beta_{i}x_{i}+\overset{k}{\underset{i=1}{\sum }}\beta_{ii}x_{i}^{2}+\sum \sum \beta_{ij}x_{i}x_{j}, \end{array}$$

where *y* is the predicted response, *β*_0_ is a constant, *x*_*i*_ and *x*_*j*_ are independent variables, and *β*_*i*_, *β*_*ii*_, and *β*_*ij*_ are the linear, quadratic, and interactive coefficients of the model, respectively.

The experimental results were analyzed using Statistic® software version 12.0, and the coefficients were interpreted using the* F *test. Three main tools were used in data analysis: analysis of variance (ANOVA), regression analysis, and a response surface plot. Effects were considered significant for a *p* value < 0.05.

## 3. Results and Discussion

### 3.1. Preliminary Tests for the HPLC-UV Method Development

**Figure 1:**
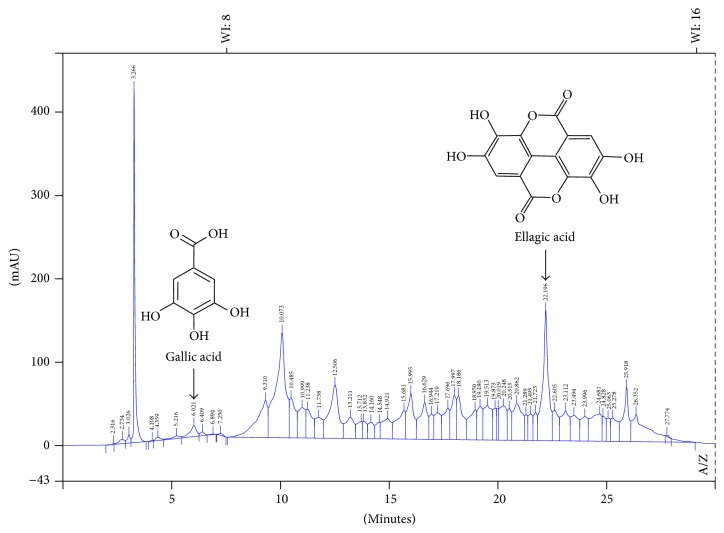
Chromatographic profile of* E. uniflora *leaves. Chromatographic conditions: Supelco Analytical C18 column (250 × 4.6 mm, 5 *μ*m); mobile phase water (A)/acetonitrile (B) (0 min 98% A and 2% B, 5 min 95% A and 5% B, 12 min 80% A and 20% B, 15 min 75% A and 25% B, 18 min 60% A and 40% B, 25 min 20% A and 80% B, 28 min 95% A and 5% B, and 30 min 98% A and 2% B); flow rate of 1.0 mL min^−1^; *λ* = 280 nm; and injection volume of 10 *μ*L.

Among the seven standards tested, gallic acid (6.26 minutes) and EA (22.84 minutes) were identified in the ethanol extract (Figure 1).

The EA peak was selected as a chemical marker for the acquisition of quality parameters of the plant material. The peak area was considered as a parameter in the marker election, and larger areas are related to higher concentrations of the analyte. Therefore, the results are indicative that EA would be one of the main constituents present in the extract.

EA is known to present low solubility in water and, especially at lower pH, the most phenolic compound is not ionized; therefore the use of alcoholic solutions to optimize the solubilization and extraction of this compounds is recommended [12, 41]. Bala et al. found an aqueous solubility value of 9.7 *μ*g mL^−1^ for EA; the value for methanol solubility was higher (671 *μ*g mL^−1^) [42].

Preliminary tests indicated that higher intensities and peak areas were obtained by using 50% ethanol (w/w). İlbay et al. evaluated different alcohol contents for the extraction of phenolic compounds from* Rosa canina* L. leaves [29]. They found the optimal conditions for the extraction included an ethanol content approximately 40% (v/v). Similar data were reported for the extraction of tannins from* Dipteryx alata* Vogel fruits [30]. As EA is a product of the hydrolysis of ellagitannins, it is believed that a lower alcohol content would favor the extraction of these components.

### 3.2. Chromatographic Conditions

**Figure 2:**
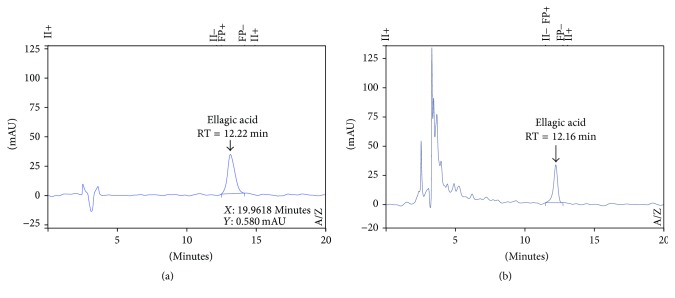
HPLC-UV chromatograms from ellagic acid analytical standard (a) and* E. uniflora* leaf extract (b) using the validated conditions: Supelco Analytical C18 column (250 × 4.6 mm, 5 *μ*m); mobile phase water/acetonitrile (85 : 15); flow rate of 1.0 mL min^−1^; *λ* = 254 nm; and injection volume of 10 *μ*L. RT = retention time.

The detection wavelength was optimized to 254 nm according to the maximum absorption wavelength of EA as reported in the literature [34, 42]. Among the evaluated chromatographic conditions, the increase in the initial ratio of acetonitrile in the elution gradient from 2% to 15% contributed to a higher elution capacity of the mobile phase and caused many unidentified peaks to coelute in the dead volume of the methodology. This change made the methodology more specific for the evaluation of the EA peak (Figures 2(a) and 2(b)).

It was observed that prolonging the duration of the gradient, which has the elution of the EA peak (60 : 40 water/acetonitrile) in agreement with a reduced flow of 1.0 to 0.5 mL min^−1^, showed significant improvement in the resolution between the peaks. This balance reached between the flow and mobile phase proportion was maintained at 1.0 mL min^−1^ and 15% of acetonitrile. As this is the initial proportion of the gradient methodology, an isocratic method was proposed for 20 minutes in a proportion of 85 : 15 (water/acetonitrile) and at a flow rate of 1.0 mL min^−1^. The resolution between the peaks was maintained (Figure 2(b)).

The use of acetonitrile in liquid chromatography as organic solvent is mainly encouraged due to its lower absorption interference at low wave-lengths compared to other solvents as methanol, and in the EA elution it reacts similar to solvent chosen for the plant extraction. For chromatographic purposes reaching an adequate analyte partition relation or analyte distribution balance during the elution is needed. This concept is brought in light by the retention factor, also known as capacity factor (*k*′). In this study, the adequate *k*′ value for EA was acquired by the proportion of organic solvent and the acidification of the mobile phase [42, 43].

To enhance the peak resolution and asymmetry (tailing) of the EA peak, three different acidifying agents (orthophosphoric acid, trichloroacetic acid, and trifluoroacetic acid) were tested in the mobile phase in the isocratic method. Among them, trichloroacetic acid (0.05%, w/v) showed greater impact on the peak resolution and asymmetry parameters.

### 3.3. Solid Phase Extraction (SPE)

SPE allowed the extraction of the vegetable matrix components without influencing the evaluation of EA. A difference in extract color indicating a greater purification was observed. Because the SPE process allows the same EA quantification in a purified extract and may improve the analytical column utilization, it was incorporated into the methodology for the preparation of the sample of liquid extract.

The chromatogram obtained from the* E. uniflora* leaf extract after SPE, using the HPLC-UV developed conditions, is presented in Figure 2(b).

### 3.4. HPLC-UV Method Validation

The validation methodology followed the sample preparation with a drug/solvent ratio of 10% (w/v) using 50% ethanol (v/v) as the extraction solvent. The extract was subjected to an ultrasound bath for 30 minutes at room temperature, and SPE has been incorporated into the end of the process. The following chromatographic conditions were validated: Supelco Analytical C18 column (250 × 4.6 mm, 5 *μ*m); mobile phase water/acetonitrile (85 : 15) acidified to 0.05% (w/v) with trichloroacetic acid; flow rate of 1.0 mL min^−1^; wavelength of 254 nm; and injection volume of 10 *μ*L.

No interference was observed by coelution of the diluent or mobile phase at the same retention time of EA at 254 nm, demonstrating the selectivity of this methodology.

The method was linear in the range of 14.5 to 33.8 *μ*g mL^−1^. The analytical curve presented a linear correlation coefficient (*r*) of 0.994 and a determination coefficient (*r*^2^) of 0.988. These data are in accordance with the specifications adopted [38].

The limits found, based on three analytical curves of the EA standard, were 0.66 *μ*g mL^−1^ for the LOD and 2.22 *μ*g mL^−1^ for the LOQ. The LOD determined from the linear regression calculation is considered as the lowest detectable concentration, but it is not necessarily quantifiable. The lowest measurable concentration of the analyte in a solution is translated in the value of the LOQ [44].

In this study, the precision data were expressed by determining the relative standard deviation (RSD) obtained by the ratio between the standard deviation of the data and the average. The RSD value for the areas of EA in the repeatability test was 3.51%. The RSD between the data of two days was 4.46%. These results were also in accordance with the specifications (RSD < 15%) [39].

The methodology showed accuracy, represented in recovery values between 105.13% and 110.61% for the test. These data are in agreement with the specification adopted for tests in complex matrices (80–120%) [37].

In the evaluation of the robustness, all of the RSDs remained below 5%, and the percentage of the assessment of the averages in relation to the normal conditions varied between 73.30 and 107.32%, whereas for a variation between 90 and 110% compared to the normal conditions, the methodology showed no robustness to a variation of −10% in its injection volume.

### 3.5. Effects of the Ultrasound-Assisted Extraction Parameters on the EA Concentration

The measurement of EA was determined based on a calibration curve (*r* = 0.99841, *y* = 64680*x* − 23.970) obtained with the validated methodology. The EA concentration data of the 15 experiments generated by the Box Behnken (3^3^) design are presented in Table 2. The concentrations of EA varied between 8.0 and 26.3 expressed in *μ*g mL^−1^.

The model presented a *p* value of 0.000358, showing no significance to the lack of fit (*p* = 0.097575). A summary of the effects is shown in Table 3. The central idea of the analysis of variance (ANOVA) is to compare the variances of the different treatments due to the variance of experimental error and thereby determine the significance of the regression adopted to provide responses [27].

As the extraction of phenolic compounds depends largely on the polarity of the solvent, it is possible that a single solvent is not efficient for the extraction of bioactive compounds [25, 42–45]. In this work, the alcohol content of the extraction solution showed a negative linear effect on the concentration of EA, indicating that lower degrees of alcohol would provide greater extraction of the marker for the leaves of* E. uniflora*. This reaffirms the preliminary assessment of the behavior of the alcohol content in the development of the analytical methodology and also showed agreement with the data of other studies [27–30].

**Figure 3:**
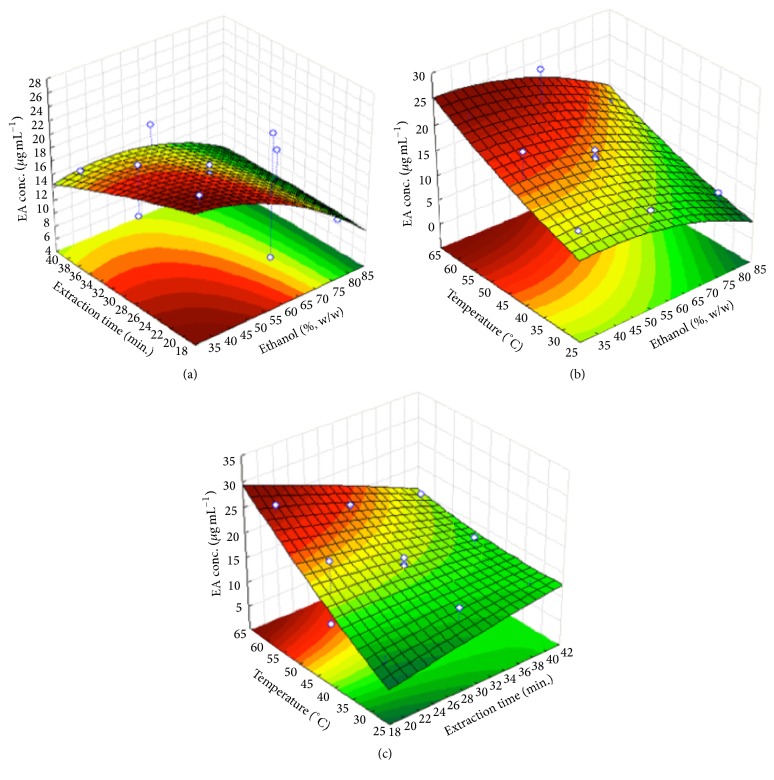
Response surface for the EA concentration from UAE experiments of* E*.* uniflora *leaves. (a) Effects of the extraction time (min) and ethanol content (%, w/w). (b) Effects of the temperature (°C) and ethanol content (%, w/w). (c) Effects of the temperature (°C) and extraction time (min).

The linear effects of the independent variables time and extraction temperature were significant as well as the interaction between them (Figure 3(c)).

With the exception of the extraction temperature, the other variables showed negative effects on the concentration of EA, as shown in the response surface graphics illustrated in Figure 3. The temperature can impact the extractive processes, leading to an increase in the substances extraction while causing losses of those that are thermosensitive [31]. It is possible that there is a higher release of EA among other phenolic compounds from the hydrolysis of the ellagitannins accentuated by temperature, which may explain the effect of this variable on the concentration of the marker [28].

Based on the analysis of response surface data using general function optimization, the UAE conditions for maximizing EA extraction were determined. Thus, the following extraction parameters have been suggested: alcohol content of 44% (w/w), 22 minutes of extraction time, and temperature of 59°C for a predicted value of 26.0 *μ*g mL^−1^ of EA. These conditions were confirmed from extractions conducted in triplicate, and the concentration of EA in relation to the predicted value was 93.7%  ±  0.4.

## 4. Conclusions

A HPLC-UV method has been developed and validated for quantifying EA in* E. uniflora* ethanol extracts. In obtaining the liquid extract by UAE, the optimization study has been shown to be able to predict responses to the levels of the variables studied. An EA concentration of 93.7 ± 0.4% compared to the predicted value under the optimized conditions (44% alcohol content (w/w), 22 minutes of extraction time and temperature of 59°C) was found. The UAE method presented a strong potential as a methodology for the extraction of EA from the leaves of* E. uniflora*.

## Figures and Tables

**Table 1:** Levels of variables for the Box Behnken (3^3^) experimental design.

Symbols	Independent variables	Levels		
		−1	0	+1
*x*_1_	Ethanol (%, w/w)	40	60	80
*x*_2_	Temperature (°C)	30	45	60
*x*_3_	Extraction time (minutes)	20	30	40

*x*_1_  = ethanol; *x*_2_  = temperature; *x*_3_  = extraction time.

**Table 2:** Box Behnken (3^3^) experimental design and concentrations of ellagic acid (EA) for the UAE of *E. uniflora* leaves.

	*x*_1_ (%)	*x*_2_ (°C)	*x*_3_ (min)	EA (*μ*g mL^−1^)
(1)	40	20	45	21.7
(2)	80	20	45	9.2
(3)	40	40	45	14.5
(4)	80	40	45	8.1
(5)	40	30	30	13.1
(6)	80	30	30	8.7
(7)	40	30	60	20.6
(8)	80	30	60	14.5
(9)	60	20	30	8.0
(10)	60	40	30	11.1
(11)	60	20	60	26.3
(12)	60	40	60	17.3
(13)	60	30	45	15.0
(14)	60	30	45	14.6
(15)	60	30	45	16.4

*x*_1_  = ethanol; *x*_2_  = temperature; *x*_3_  = extraction time.

**Table 3:** Summary of the effects of factors and their significance (*p*  value).

Factor	*p* value	Effect
*x*_1_	0.008167^*∗*^	−0.00735
*x*_2_	0.074161^*∗∗*^	0.001704
*x*_3_	0.033663^*∗*^	−0.00355
*x*_1_^2^	0.65681	0.000254
*x*_2_^2^	0.004965^*∗*^	0.00945
*x*_3_^2^	0.349469^*∗*^	−0.000596
*x*_1_ *x*_2_	0.084095^*∗∗*^	0.00305
*x*_1_ *x*_3_	0.463396	−0.00085
*x*_2_ *x*_3_	0.023548^*∗*^	−0.00605

*x*_1_  = ethanol; *x*_2_  = temperature; *x*_3_  = extraction time; ^*∗*^*p* < 0.05; ^*∗∗*^*p* < 0.1.
